# Homologous desensitization of signalling by the alpha (α) isoform of the human thromboxane A_2_ receptor: A specific role for nitric oxide signalling

**DOI:** 10.1016/j.bbamcr.2007.03.012

**Published:** 2007-06

**Authors:** Leanne P. Kelley-Hickie, Martina B. O'Keeffe, Helen M. Reid, B. Therese Kinsella

**Affiliations:** School of Biomolecular and Biomedical Science, Conway Institute of Biomolecular and Biomedical Research, University College Dublin, Belfield, Dublin 4, Ireland

**Keywords:** C-tail, carboxyl-terminal tail, [Ca^2+^]_i_, intracellular calcium, COX, cyclooxygenase, FBS, foetal bovine serum, GPCR, G protein-coupled receptor, GRK, G protein-coupled receptor kinase, HA, hemagglutinin, HEK, human embryonic kidney, IP, prostacyclin receptor, IP_3_, inositol 1, 4, 5-trisphosphate, NO, nitric oxide, NOS, nitric oxide synthase, PAGE, polyacrylamide gel electrophoresis, PG, prostaglandin, PK, protein kinase, PL, phospholipase, sGC, soluble guanylyl cyclase, TP, TXA_2_ receptor, TX, thromboxane, Thromboxane receptor, Alpha, Desensitization, Phosphorylation, Nitric oxide, G protein coupled receptor

## Abstract

Thromboxane (TX) A_2_ plays a central role in hemostasis, regulating platelet activation status and vascular tone. We have recently established that the TPβ isoform of the human TXA_2_ receptor (TP) undergoes rapid, agonist-induced homologous desensitization of signalling largely through a G protein-coupled receptor kinase (GRK) 2/3-dependent mechanism with a lesser role for protein kinase (PK) C. Herein, we investigated the mechanism of desensitization of signalling by the TPα isoform. TPα undergoes profound agonist-induced desensitization of signalling (intracellular calcium mobilization and inositol 1,4,5 trisphosphate generation) in response to the TXA_2_ mimetic U46619 but, unlike that of TPβ, this is independent of GRKs. Similar to TPβ, TPα undergoes partial agonist-induced desensitization that occurs through a GF 109203X-sensitive, PKC mechanism where Ser^145^ within intracellular domain (IC)_2_ represents the key phospho-target. TPα also undergoes more profound sustained PKC- and PKG-dependent desensitization where Thr^337^ and Ser^331^, respectively, within its unique C-tail domain were identified as the phospho-targets. Desensitization was impaired by the nitric oxide synthase (NOS), soluble guanylyl cyclase (sGC) and PKG inhibitors l-NAME, LY 83583 and KT5823, respectively, indicating that homologous desensitization of TPα involves nitric oxide generation and signalling. Consistent with this, U46619 led to rapid phosphorylation/activation of endogenous eNOS. Collectively, data herein suggest a mechanism whereby agonist-induced PKC phosphorylation of Ser^145^ partially and transiently impairs TPα signalling while PKG- and PKC-phosphorylation at both Ser^331^ and Thr^337^, respectively, within its C-tail domain profoundly desensitizes TPα, effectively terminating its signalling. Hence, in addition to the agonist-mediated PKC feedback mechanism, U46619-activation of the NOS/sGC/PKG pathway plays a significant role in inducing homologous desensitization of TPα.

## 1 Introduction

The prostanoid thromboxane (TX)A_2_, synthesized through the sequential actions of cyclooxygenase (COX) 1 and/or COX 2 and TXA_2_ synthase, plays a critical role in the dynamic control of hemostasis and vascular tone [1,2] and may induce other diverse cellular responses including mitogenic and/or hypertrophic growth of vascular smooth muscle [3–5], inhibition of angiogenesis/neo-vascularization [6–8] and apoptosis of CD4/CD8^+/−^ immature thymocytes [9]. Additionally, the TXA_2_ receptor (TP) mediates at least some of the adverse actions of the isoprostane 8-iso prostaglandin (PG)F_2α_, generated in situations of oxidative stress [10,11]. In keeping with these actions, imbalances in the levels of TXA_2_ or of its synthase or receptor (TP) have been implicated in a number of vascular diseases including thrombosis, unstable angina, bronchial asthma, hypertension and glomerulonephritis [3–5,12,13].

The TXA_2_ receptor, or TP, a member of the G protein coupled receptor superfamily, is primarily coupled to Gq/phospholipase (PL)Cβ activation and to mobilization of Ca^2+^ from inositol 1,4,5 trisphosphate-operated intracellular stores [1]. In humans, TXA_2_ signals through two TP isoforms termed TPα and TPβ that are identical for their N-terminal 328 amino acids differing exclusively in their carboxyl terminal (C)-tail domains [14–16]. Whilst the significance of two receptors for TXA_2_ in humans, but not in non-primates, is unknown there is substantial evidence that they display critical differences in their signalling and profiles of expression and hence it is thought likely that they have distinct physiologic/pathophysiologic roles [17]. TPα and TPβ exhibit distinct patterns of expression in a variety of cell/tissue types of vascular origin, including platelets [18–20] and such differences are due to the fact that TPα and TPβ are not only products of differential splicing but are also under the transcriptional control of distinct promoters within the single human TP gene [21–24]. While TPα and TPβ exhibit identical ligand binding and coupling to PLCβ [15,25,26], they oppositely regulate adenylyl cyclase [27] and TPα, but not TPβ, mediates activation of Gh, leading to PLC activation [28].

A central feature of the general GPCR signal transduction cascade is the regulation or desensitization of second messenger generation and signalling that occurs in response to the continued presence of the ligand, dampening or terminating the specific cellular response [29,30]. Such desensitization is typically initiated by GPCR phosphorylation leading to uncoupling of the receptor from its cognate G-protein [29,30] and may involve either cross-talk/heterologous desensitization between different signalling systems or agonist-induced/homologous desensitization terminating or modulating the response to the receptor's own ligand [29,30]. In studies investigating heterologous desensitization or cross-talk between TXA_2_ and other prostanoids, it has been recently established that while both TP isoforms have evolved to share a similar mechanism of protein kinase (PK)C-induced phosphorylation in response to signalling by the EP_1_ subtype of the prostaglandin (PG) E_2_ receptor and by the PGF_2α_ receptor (FP), those phosphorylations/desensitizations occur at distinct PKC sites within the unique C-tail domains of TPα and TPβ, respectively [31,32]. On the other hand, signalling by TPα, but not TPβ, is subject to prostacyclin and PGD_2_-mediated desensitization in a mechanism involving direct protein kinase (PK) A phosphorylation of TPα at Ser^329^, the very first residue within its unique C-tail domain divergent from TPβ [33,34]. Consistent with the latter, TPα, but not the TPβ, is also a target for nitric oxide (NO)-induced heterologous desensitization of signalling that occurs through direct cGMP-dependent PKG/cGK phosphorylation at Ser^331^ also within its C-tail [35]. Collectively, these latter studies indicate critical differences in the modes of regulation of signalling by the individual TP isoforms. Specifically, they point to an essential role for TPα in prostacyclin- and NO-regulated vascular hemostasis and point to a redundant or an, as yet, unidentified role for TPβ in this essential physiologic process [33–35].

As stated, agonist-induced homologous desensitization of GPCRs is a central mechanism whereby the cellular responses to the receptor's own ligand is dynamically modulated and is most often initiated through phosphorylation of the ligand-engaged, conformationally active GPCR such as by members of the G protein-coupled receptor kinases (GRKs) and/or by the second-messenger kinases, such as PKA or PKC [29,30,36]. While both TP isoforms undergo rapid agonist-induced phosphorylation in response to TXA_2_ mimetic U46619 [20,25], TPβ, but not TPα, is subject to GRK/β-arrestin-dependent internalization following prolonged agonist exposure [37,38]. We recently examined the mechanism of agonist-induced homologous desensitization of signalling and second messenger generation by TPβ and established that it undergoes two main mechanisms of desensitization: (i) partial, transient PKC-dependent desensitization where Ser^145^ within IC_2_ was identified as the PKC phospho-target. In addition, (ii) TPβ also undergoes more profound and sustained agonist-induced desensitization involving GRK 2/3-phosphorylation followed by internalization and we proposed a model whereby phosphorylation of Ser^357^ within the C-tail domain led to the recruitment and orientation of GRK2/3 which, in turn, facilitated its phosphorylation of Ser^239^ within IC_3_ thereby disrupting Gq coupling by TPβ. Hence, GRK2/3 binding is dependent on sequences within the unique C-tail domain of TPβ whilst Gq uncoupling is due to GRK phosphorylation of Ser^239^ within IC_3_ and, to a lesser extent, PKC phosphorylation of Ser^145^ within IC_2_.

In view of these findings, the aim of the current studies was to investigate the mechanism of homologous desensitization of signalling by TPα in response to the TXA_2_ mimetic U46619. Our data established that TPα is subject to homologous desensitization of signalling through two key mechanisms involving receptor phosphorylation: one involving a partial PKC-dependent mechanism and another involving a profound and sustained PKG-dependent mechanism engaging NO signalling and endothelial (e) nitric oxide synthase (NOS) activation. Unlike that of TPβ, we found no role for the GRK/β-arrestins in homologous desensitization of TPα. Hence, taken together, these studies demonstrate critical differences in the mechanisms of agonist-induced desensitization of signalling between the TP isoforms and add to the increasing lines of evidence for distinct physiologic roles for TPα and TPβ in the mediation and the regulation of signalling by the potent autocoid TXA_2_ in humans.

## 2 Materials and methods

### 2.1 Materials

SQ29,548 and I-BOP were from the Cayman Chemical Company. Anti-Gαq (C15; SC-392) antibody was obtained from Santa Cruz Laboratories. [^32^P] orthophosphate (8000–9000 Ci/mmol) and [^3^H]SQ29,548 (50.4 Ci/mmol) was from NEN Life Sciences Products. [^3^H] IP_3_ (20–40 Ci/mmol) was obtained from American Radiolabelled Chemicals Inc. Anti-HA 101R monoclonal antibody and anti-HA-3F10-peroxidase conjugated antibody were obtained from BABCO and from Roche Molecular Biochemicals, respectively. Monoclonal anti-eNOS antibody (Clone 3) was obtained from Transduction Laboratories. Anti-phospho-eNOS^Ser1177(Human)/1179(Bovine)^ antibody was purchased from Cell Signalling Technology. Thrombin was obtained from Sigma Chemical Co. FURA2/AM and d-*myo*-inositol 1,4,5-trisphosphate, 3-deoxyhexasodium salt, U46619, *N*-[2-((*p*-bromocinnamyl)amino)ethyl]-5-isoquino-linesulfonamide, 2HCL (H-89) and 2-[1-(3-dimethylaminopropyl)-1H-indol-3-yl]-3-(1H-indol-3-yl)-maleimide (GF 109203X), NG-Nitro-l-arginine Methyl Ester, Hydrochloride (l-NAME), KT 5823, LY 83583 (6-Anilino-5, 8-quinolinequinone) were obtained from Calbiochem.

### 2.2 Subcloning and site-directed mutagenesis

The plasmids pCMV:Gαq, pHM:TPβ, pHM:TPα, pHM:TP^Δ328^, pHM:TPα^S329A^, pHM:TPα^S331A^, pHM:TPα^T337A^, pHM:TPα^S329,331A^, pHM:TPα^Δ336^ and pRK5:βARK1^495–689^ have been previously described [10,32,33,35,39].

**Fig. 1:**
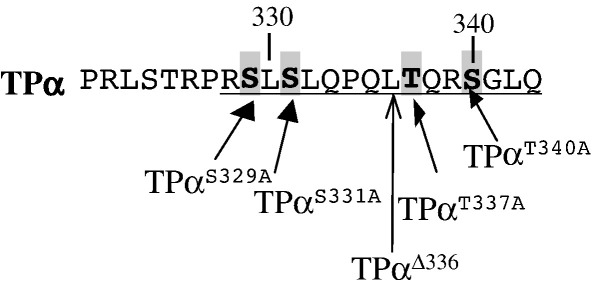
Schematic of the carboxyl (C) tail domain of TPα. The amino acid sequence of the carboxyl terminal (C)-tail domain of TPα (residues 321–343) is shown, where residues unique to TPα (residues 329–343) are underlined. The truncation (Δ) mutant TPα^Δ336^, generated by conversion of Leu^336^ codon to a stop codon, is indicated by the open arrow head while Ser/Thr to Ala substitutions to generate TPα^S329A^, TPα^S331A^, TPα^T337A^ and TPα^T340A^ mutations are indicated by the solid arrows. The combination substitutions TPα^S329,331A^, TPα^S331,T337A^, TPα^S331,340A^, TPα^T337,S340A^ and TPα^S331,T337,S340A^ were also generated. Mutations involving Ser^145^ or Ser^239^ within IC_2_ or IC_3_, respectively, either alone or in combination with the C-tail mutations are not shown.

Fig. 1 illustrates the C-tail sequence of TPα and its mutated variants that were generated and used in this study. Site-directed mutagenesis was performed using QuickChange^TM^ (Stratagene), unless specified otherwise. Mutation of Ser^145^ to Ala^145^ of TPα and TP^Δ328^ to generate the plasmids pHM:TPα^S145A^ and pHM:TP^S145A, Δ328^ was achieved using pHM:TPα and pHM:TP^Δ328^, respectively, as templates and sense/antisense primer pair (5′-GC CCG GCG GTC GCC ***GCG*** CAG CGC GCC-3′). Mutation of Ser^239^ to Ala^239^ of TPα to generate pHM:TPα^S239^ was achieved using pHM:TPα as template and sense/antisense primer pair (5′-G CGT CCC CGG GAC ***GCC*** GAG GTG GAG A-3′). Conversion of both Ser^331^,Thr^337^ to Ala^331^,Ala^337^of TPα to generate pHM6: TPα^S331,337A^ was achieved using pHM:TPα^T337A^ as template and sense/antisense primer pair (5′-G CCC AGG TCG CTG ***GCC*** CTC CAG CCC C-3′). Mutation of Ser^340^ to Ala^340^ of TPα, TPα^S331A^ and TPα^T337A^ to generate pHM:TPα^S340A^, pHM:TPα^S331,340A^ and pHM:TPα^T337,S340A^ was achieved using pHM:TPα, pHM:TPα^S331A^ and pHM:TPα^T337A^, respectively, as templates and sense/antisense primer pair (5′-C ACG CAG CGC ***GCC*** GGG CTG CAG TAG G-3’). Mutation of Ser^340^ to Ala^340^ of TPα^S331,T337A^ to generate pHM:TPα^S331,T337,S340A^ was achieved using pHM:TPα^S331,T337A^ as template and sense/antisense primer pair (5′-CAG CCC CAG CTC ***GCG*** CAG CGC GCC G-3′). Mutation of Ser^145^ to Ala^145^ of TPα^S331,T337A^ to generate pHM:TPα^S145,S331,T337A^ was achieved using pHM:TPα^S331,T337A^ as template and sense/antisense primer pair (5′-GC CCG GCG GTC GCC ***GCG*** CAG CGC GCC-3′). For each primer pair above, sequence shown corresponds to the sense primer and in each case the identity of the mutator codon is in boldface italics.

### 2.3 Cell culture and transfections

Human embryonic kidney (HEK) 293 cells were cultured in minimal essential medium with Earle's salts (MEM) supplemented with 10% FBS (foetal bovine serum) and maintained at 37 °C in 5% CO_2_. The following HEK 293 cell lines stably over-expressing hemagglutinin (HA) epitope-tagged forms of TPβ (HEK.TPβ),  TPα (HEK.TPα),  TP^Δ328^ (HEK.TP^Δ328^), TPα^S329A^ (HEK.TPα^S329A^), TPα^S337A^ (HEK.TPα^T337A^), TPα^S331A^ (HEK.TPα^S331A^), TPα^S329,331A^ (HEK.TPα^S329,331A^) and TPα^Δ336^ have been previously described [32,33,35].

For transfections, routinely HEK 293 cells were plated in 10 cm dishes at a density of 2 × 10^6^ cells/dish in 8 ml media 48 h prior to transfection. Cells were transiently transfected with 10 μg pADVA [40] and 25 μg of pcDNA-, pCMV- or pHM-based vectors using the calcium phosphate/DNA co-precipitation procedure as previously described [10]. For transient transfections, cells were harvested 48 h post transfection. To create HEK 293 cell lines stably over-expressing HA-epitope tagged forms of TPα^S145A^ (HEK.TPα^S145A^),  TP^S145A,Δ328^ (HEK.TP^S145A,Δ328^), TPα^S239A^ (HEK.TPα^S239A^),  TPα^S340A^ (HEK.TPα^S340A^), TPα^S331,T337A^ (HEK.TPα^S331,T337A^), TPα^S331,340A^ (HEK.TPα^S331,340A^),  TPα^T337,S340A^ (HEK.TPα^T337,S340A^), TPα^S331,T337,S340A^ (HEK.TPα^S331,T337,S340A^),  TPα^S134,S331,T337 A^ (HEK.TPα^S145,S331,T337A^),  cells were transfected with 10 μg of Sca1-linearised pADVA plus 25 μg of the appropriate Pvu1-linearised pHM6-based recombinant plasmids. Forty-eight hours post-transfection, G418 (0.8 mg/ml) was applied and after approximately 21 days, individual G418-resistant colonies were selected and individual pure clonal stable cell lines/isolates were examined for TP expression by analysis of radioligand binding.

### 2.4 Radioligand binding studies

Cells were harvested by centrifugation at 500×*g* at 4 °C for 5 min and washed three times with ice-cold Ca^2+^/Mg^2+^-free phosphate-buffered saline (PBS). TP radioligand binding assays were carried out at 30 °C for 30 min in 100 μl reactions in the presence of 0–40 nM [^3^H] SQ29,548 for Scatchard analysis or in the presence of 20 nM [^3^H] SQ29,548 for saturation radioligand binding experiments as previously described [10]. Protein determinations were carried out using the Bradford assay [41].

### 2.5 Measurement of intracellular calcium ([Ca^2+^]_i_) mobilization

Measurement of intracellular calcium mobilization ([Ca^2+^]_i_) in FURA2/AM preloaded HEK 293 cell lines (2 × 10^6^ cells/dish), each transiently co-transfected with pADVA (10 μg), and pCMV:Gαq (25 μg), was carried out as previously described [10]. To investigate the effect of GRK2/βARK1 on TP-mediated [Ca^2+^]_i_ mobilization, HEK.TPα or HEK.TPβ cells (2 × 10^6^ cells/dish) were co-transfected with pADVA (10 μg), pCMV:Gαq (25 μg) along with pRK5:βARK1^495–689^ (25 μg), encoding a dominant negative form of GRK2/βARK1[39]. Approximately 48 h post-transfection, cells were harvested by scraping, washed twice in ice-cold PBS and resuspended in HBSSHB (modified Ca^2+^/Mg^2+^-free Hank's buffered salt solution, containing 10 μM HEPES, pH 7.67, 0.1% bovine serum albumin (BSA)) buffer at 10^7^ cells/ml and incubated in the dark with 5 μM FURA2/AM for 45 min at 37 °C. Cells were collected by centrifugation (900×*g*, 5 min), washed once in an equal volume of HBSSHB, and were finally resuspended in HBSSHB buffer at 10^7^ cells/ml and kept at room temperature in the dark for 40 min. For each measurement of [Ca^2+^]_i_ mobilization, aliquots of cells were diluted to 0.825 × 10^6^ cells/ml in HBSSHB, 1 mM CaCl_2_ and FURA2 fluorescence was recorded (2 ml aliquots of cells) at 37^ ^°C with gentle stirring with a Perkin Elmer-Cetus LS50-B spectrofluorometer at excitation wavelengths of 340 nm and 380 nm and emission wavelength of 510 nm, respectively.

Cells were stimulated with the TP agonist U46619 (1 μM) and changes in [Ca^2+^]_i_ mobilization were monitored as a function of time. To assess agonist-mediated homologous desensitization, cells (0.825 × 10^6^ cells/ml; 2 ml per assay) were stimulated with 1 μM U46619 (Primary stimulation) or, as a control, with the vehicle for 4 min; thereafter, cells were collected by centrifugation (900×*g*, 5 min); were washed twice in 2 ml HBSSHB, 1 mM CaCl_2_; and finally resuspended in 2 ml HBSSHB,1 mM CaCl_2_ and were left to recover over various time intervals (ranging from 0 to 60 min); thereafter, cells re-stimulated with 1 μM U46619 (Secondary stimulation) and changes in [Ca^2+^]_i_ mobilization were monitored as a function of time. To assess the role of protein kinase (PK) A, PKC or PKG on U46619-mediated desensitization of TPα signalling, cells were pre-incubated with the PKA inhibitor H-89 (10 μM), the PKC inhibitor GF 109203X (50 nM) or the PKG inhibitor KT 5823 (50 nM), for 15 min and respective kinase inhibitors were maintained throughout the primary/secondary U46619-stimulations and during the washes. To assess the role of the nitric oxide (NO) pathway on U46619-mediated desensitization of TPα signalling, cells were pre-incubated with the NO synthase inhibitor l-NAME (1 μM) or the soluble guanylate cyclase (sGC) inhibitor LY 83583 (1 μM) for 15 min and respective inhibitors were maintained throughout the primary/secondary U46619-stimulations and during the washes.

In all cases, the drugs (agonist or inhibitors in 0.01% ethanol in HBS) were diluted in the vehicle HBSSHB such that addition of 20 μl of the diluted drug/inhibitor to 2 ml of cells resulted in the correct working concentration. The vehicle had no effect on U46619-mediated [Ca^2+^]_i_ mobilization. Calibration of the signal was performed in each sample by adding 0.2% Triton X-100 to obtain the maximal fluorescence ratio (*R*_max_) and then 1 mM EGTA to obtain the minimal fluorescence ratio (*R*_min_). The ratio of the fluorescence at 340 nm to that at 380 nm is a measure of [Ca^2+^]_i_ [42], which assumes a *K*_d_ of 225 nM Ca^2+^ for FURA2/AM. The results presented in the figures are representative data from at least three or four independent experiments and are plotted as changes in Ca^2+^_i_ mobilized (Δ[Ca^2+^]_i_ (nM)) as a function of time (s) upon ligand stimulation. Changes in [Ca^2+^]_i_ mobilization were determined by measuring peak rises in [Ca^2+^]_i_ mobilized (Δ[Ca^2+^]_i_) and were calculated as mean changes in Δ[Ca^2+^]_i_ ± S.E.M. (nM).

### 2.6 Measurement of IP_3_ levels

Intracellular IP_3_ levels were measured as previously described [33,43]. Briefly, cells were harvested by scraping, washed twice in ice-cold PBS and resuspended at approximately 2 × 10^6^ cells/200 μl in HEPES-buffered saline (HBS; 140 nM NaCl, 4.7 mM KCl, 2.2 mM CaCl_2_, 1.2 mM KH_2_PO_4_, 11 mM glucose, 15 mM HEPES–NaOH, pH 7.4), 10 mM LiCl (Walsh et al., 2000). Cells (200 μl) were pre-incubated in HBS buffer at 37 °C for 10 min; where appropriate inhibitors (H-89, 10 μM; GF 109203X, 50 nM; KT 5823, 50 nM; l-NAME, 1 μM; LY 83583, 1 μM) or vehicle (HBS) were added to the cells and pre-incubated at 37 °C for 15 min. Thereafter, cells were stimulated with 1 μM U46619 at 37 °C for 2 min. To assess agonist-mediated homologous desensitization, cells (2 × 10^6^ cells/200 μl) were stimulated with 1 μM U46619 at 37 °C for 2 min (Primary stimulation); thereafter, cells were collected by centrifugation (900×*g*, 5 min); were washed once in 200 μl HBS, 10 mM LiCl and finally resuspended in 200 μl HBS, 10 mM LiCl and were left to recover for 60 min; thereafter, cells were re-stimulated with 1 μM U46619 at 37 °C for 2 min (secondary stimulation). All ligands and kinases were pre-diluted in HBS such that 50 μl added to 200 μl of cell suspension in HBS gave the desired final concentration. To determine basal IP_3_ levels, an equivalent volume (50 μl) of the vehicle HBS was added instead of ligand. The level of IP_3_ produced was quantified by radio competition assay essentially as described [43]. Levels of IP_3_ produced by ligand stimulated cells over basal stimulation, in the presence of HBS, were expressed in pmol IP_3_/mg protein ± S.E.M. and as fold stimulation over basal (fold increase ± S.E.M.). In all cases, 4 independent experiments were performed, each in duplicate.

### 2.7 Measurement of agonist-mediated TP phosphorylation

Agonist-mediated TP phosphorylation in whole cells (1.8–2 × 10^6^ cells/10 cm dish) was performed essentially as previously described [33]. Briefly, cells were transiently co-transfected with pADVA (10 μg), pCMV:Gαq (25 μg) plus pcDNA:βArrestin2 (25 μg) approximately 48 h prior to labelling. Thereafter, cells were washed once in phosphate-free DMEM, 10% dialysed FBS and were metabolically labelled for 60 min in the same medium (2 ml/10 cm dish) containing 100 μCi/ml [^32^P] orthophosphate (8000–9000 Ci/mmol) at 37 °C, 5% CO_2_. Where appropriate, the PKG kinase inhibitor KT 5823 (50 nM) or its vehicle was added during the labelling period. Thereafter, U46619 (1 μM) or an equivalent volume of vehicle (0.01% ethanol in HBS; 20 μl) were added and cells were incubated for 10 min at 37 °C, 5% CO_2_. Reactions were terminated by transferring the dishes to ice and aspirating the labelled medium and HA-epitope tagged TP receptors were processed and immunoprecipitated using the anti-HA 101R antibody as previously described [33]. Electroblots were then exposed to Xomat XAR (Kodak) film to detect ^32^P-labelled proteins. Thereafter, blots were subject to phosphorimage analysis, and the intensities of agonist-induced phosphorylation were expressed in arbitrary units relative to basal (vehicle-stimulated) levels. In parallel experiments, cells were incubated under identical conditions in the absence of [^32^P] orthophosphate; HA-tagged TP receptors were immunoprecipitated from the same cell lines using the anti-HA 101R antibody and immunoblots were screened using the anti-HA 3F10 horseradish peroxidase-conjugated antibody [33].

### 2.8 Analysis of NOS expression and eNOS phosphorylation

RNA extracted from HEK 293 cells (1.4 μg in 25 μl reactions) were converted to first strand (1°) cDNA using MMLV RT essentially as previously described [18]. Aliquots (3.5 μl) of each 1° cDNA were then used as templates in PCR reactions (25 μl) using forward (F) and reverse (R) primer pairs selective for human endothelial (*e*) nitric oxide synthase, (F-[5′-CCA GCT AGC CAA AGT CAC CAT-3′], R-[5′-GTC TCG GAG CCA TAC AGG ATT-3′], amplicon 354 bp; [44–46]); neuronal (*n)*NOS, (F-[5′-TTG GGG GCC TGG GAT TTC TGG-3′], R-[5′CGT TGG CAT GGG GGA GTG AGC-3′], amplicon 465 bp; [47]) and inducible (*i)*NOS, F-[5′-GAG GAA GTG GGC AGG AGA ATG-3′], R-[5′-GTA GTA GAA AGG GGA CAG GAC-3′], amplicon 294 bp; [48,49]). For each primer pair, PCRs carried out in the absence of template served as controls. For western analysis, aliquots of total HEK 293 cell protein were analysed by SDS-PAGE; blots were screened versus anti-eNOS antibody (Transduction Laboratories) following by chemiluminescence detection, essentially as previously described [33].

In order to assess eNOS phosphorylation, HEK.TPα, HEK.TPβ or HEK 293 cells (2 × 10^6^ cells/10-cm dish) were plated some 48 h prior to experimentation. Cells were incubated with vehicle, 1 μM U46619 or 10 U/ml Thrombin for 5 min at 37 °C and aliquots of whole cell protein (100 μg/lane) were analysed by SDS-PAGE/western blot. Blots were initially screened using the anti-phospho eNOS^1177^ as per supplier's instructions (Cell Signalling Technology) and, following stripping, were subsequently re-screened using the anti-eNOS (Transduction Laboratories).

### 2.9 Data analysis

Radioligand binding and Scatchard analysis data were analysed using GraphPad Prisim V4.0 programme (GraphPad Software Inc., San Diego CA, USA). Statistical analyses were carried out using the unpaired Student's *t* test using the Statworks Analysis Package. *p*-values ≤ 0.01 indicated statistically significant differences.

## 3 Results

### 3.1 Effect of primary and secondary agonist stimulation on TPα-signalling

We have recently investigated the mechanism of agonist-induced homologous desensitization of signalling by the TPβ isoform of the human thromboxane (TX) A_2_ receptor [50]. Herein, the aim of the current study was to investigate the mechanism of homologous desensitization of signalling by the TPα isoform of that receptor. We initially investigated desensitization of TPα signalling ([Ca^2+^]_i_ mobilization and IP_3_ generation) in response to the TXA_2_ mimetic U46619 by comparing it to that of the previously characterised TP^Δ328^ [50], a truncated variant devoid of the divergent residues within the C-tail domains of TPα and TPβ [26,33].

**Fig. 2:**
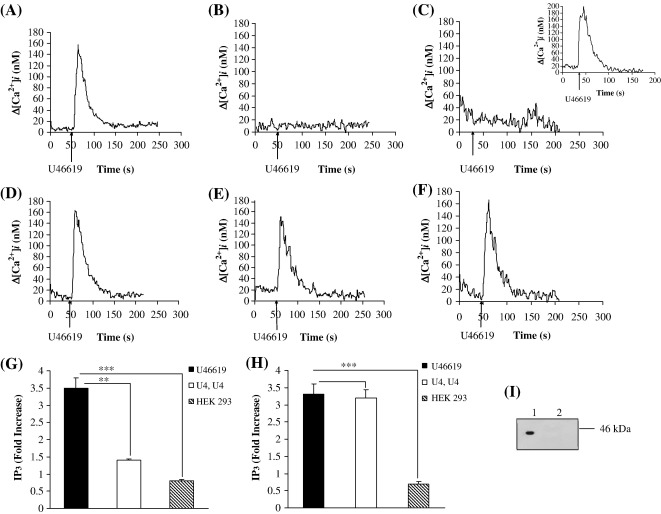
U46619-mediated desensitization of signalling by TPα. Panels A–F: HEK.TPα (Panels A–C) or HEK.TP^Δ328^ (Panels D–F) cells, transiently co-transfected with pCMV:Gαq, were stimulated with 1 μM U46619 for 4 min as primary stimulation (Panels A and D). Thereafter, cells were washed to remove the U46619 as indicated by the horizontal arrow and were then re-stimulated with 1 μM U46619 either 15 min (Panels B and E) or 60 min (Panels C and F) following the primary U46619 stimulation, where ligands were added at the times indicated by the vertical arrows. Panel C, inset: HEK.TPα cells were stimulated with vehicle for 4 min as the initial primary stimulation (data not shown) prior to washout and subsequent stimulation with 1 μM U46619 at 60 min. Data presented are plotted as changes in intracellular Ca^2+^ mobilization (Δ[Ca^2+^]_i_, nM) as a function of time (second, s). Actual mean changes in U46619-induced [Ca^2+^]_i_ mobilization (nM ± S.E.M.) were as follows: Panel A: Δ[Ca^2+^]_i_ = 174 ± 8.8 nM; Panel B: Δ[Ca^2+^]_i_ = 0 nM; Panel C: Δ[Ca^2+^]_i_ = 0 nM; Panel C inset: Δ[Ca^2+^]_i_ = 191.5 ± 25.5 nM; Panel D: Δ[Ca^2+^]_i_ = 180 ± 5.6 nM; Panel E: Δ[Ca^2+^]_i_ = 158 ± 3.8 nM; Panel F: Δ[Ca^2+^]_i_ = 178 ± 6.7 nM. Panels G and H: HEK.TPα (Panel G) or HEK.TP^Δ328^ (Panel H) cells, transiently co-transfected with pCMV:Gαq or, as controls, HEK 293 cells (Panels G and H; HEK 293) were stimulated with 1 μM U46619 for 2 min (U46619); alternatively, cells were stimulated with 1 μM U46619 for 2 min, washed to remove the agonist and re-stimulated 60 min following the primary stimulation with 1 μM U46619 for 2 min (U4,U4). Levels of IP_3_ produced in ligand-stimulated cells relative to vehicle (HBS)-treated cells (basal IP_3_) were expressed as fold stimulation of basal (fold increase in IP_3_ ± S.E.M.; *n* = 4). The asterisks indicate that the level of U46619-mediated IP_3_ generation was significantly reduced following secondary stimulation compared to that of the primary stimulation, or that the level of U46619-mediated IP_3_ generation was significantly lower in HEK 293 cells than in each of the above cell lines where ** and *** indicates *p* < 0.01 and *p* < 0.001, respectively. Basal levels of IP_3_ in HEK.TPα (Panel G), HEK.TP^Δ328^ (Panel H) and HEK 293 cells (Panels G and H) were found to be in the range of 0.27–0.39 nmol/mg protein. Panel I: HEK.TPα cells co-transfected with pCMV:Gαq (lane 1) or the control vector pCMV5 (lane 2) were analysed by SDS PAGE (75 μg whole cell protein analysed/lane) followed by western blot analysis using anti-Gαq antibody (Gα_q/11_ (C-19): S.C. 392). Data presented is a representative immunoblot from four independent experiments. The relative position of the 46-kDa molecular size marker is indicated to the right of Panel I.

Stimulation of HEK.TPα cells, stably over-expressing TPα (Table 1), with the TXA_2_ mimetic U46619 resulted in efficient mobilization of intracellular calcium ([Ca^2+^]_i,_ Fig. 2A), consistent with previous findings [26,33]. Pre-stimulation of HEK.TPα cells with U46619 almost completely desensitized TPα signalling following agonist-washout and secondary re-stimulation at 15 min following the initial U46619 stimulation (Fig. 2B, *p* = 0.0001). Moreover, U46619-desensitization of TPα signalling was sustained and did not significantly recover even at 60 min following the primary stimulation (Fig. 2C, *p* = 0.0001). On the other hand, pre-treatment of HEK.TPα cells with the vehicle, as opposed to U46619 itself, during the primary stimulation followed by washout confirmed that the cells retained the ability to fully signal in response to subsequent stimulation with U46619 (Fig. 2C, inset). Hence the loss of the secondary U46619-response was not due to mechanical stress during agonist-washout *per se* but rather resulted from agonist-induced desensitization of TPα signalling.

While primary simulation of HEK.TP^Δ328^ cells also led to efficient U46619-induced [Ca^2+^]_i_ mobilization (Fig. 2D), consistent with previous data [50] pre-stimulation with U46619 only partially desensitized [Ca^2+^]_i_ mobilization by TP^Δ328^ such that ∼ 80% of its primary U46619-response occurred following its restimulation at 15 min (Compare Fig. 2D versus Fig. 2E, *p* = 0.2). Moreover, desensitization of U46619-mediated [Ca^2+^]_i_ mobilization by TP^Δ328^ was transient such that its signalling had fully recovered showing no desensitization at 60 min (Compare Fig. 2D versus Fig. 2F; *p* = 0.22). Consistent with these data, primary stimulation of HEK.TPα and HEK.TP^Δ328^ cells with U46619 also yielded efficient increases in IP_3_ generation (Figs. 2G and H, respectively). While pre-incubation with U46619 almost completely inhibited IP_3_ generation by TPα following its secondary restimulation (Fig. 2G, *p* = 0.0049), it had no significant effect on IP_3_ generation by TP^Δ328^ at 60 min following the primary stimulation (Fig. 2H, *p* = 1). Moreover, similar agonist-induced desensitization of [Ca^2+^]_i_ mobilization and IP_3_ generation occurred when I-BOP was used as the TP-stimulatory ligand (data not shown). Taken together these data confirm that like TPβ, TPα is subject to near complete and sustained agonist-induced homologous desensitization while, consistent with previous reports [50], TP^Δ328^ undergoes partial and transient desensitization suggesting that the major target site(s) involved in homologous desensitization of TPα may be located within its unique C-tail domain.

**Fig. 3:**
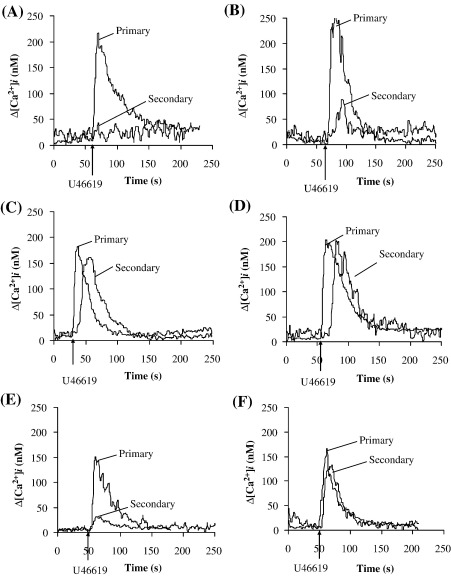
Effect of H-89 and GF 109203X on U46619-mediated desensitization of TPα signalling. Panels A–F: HEK.TPα (Panels A and B) or, as controls, HEK.TP^Δ328^ (Panels C and D) cells were pre-incubated for 10 min with either 10 μM H-89 (Panels A and C) or 50 nM GF 19203X (Panels B and D) prior to stimulation for 4 min with 1 μM U46619 (primary stimulation; Panels A, B, C and D); cells were then washed to remove the U46619 and were then re-stimulated at 15 min following the primary stimulation with 1 μM U46619 in the presence of 10 μM H-89 (Secondary stimulation; Panels A and C) or 50 nM GF 109203X (Secondary stimulation; Panels B and D). Data presented are representative profiles from at least four independent experiments and are plotted as changes in intracellular Ca^2+^ mobilization (Δ[Ca^2+^]_i_, nM) as a function of time (second, s), where ligands were added at the times indicated by the arrows. Actual mean changes in U46619-induced [Ca^2+^]_i_ mobilization (nM ± S.E.M.) in response to primary and secondary U46619-stimulations were: Panel A: Δ[Ca^2+^]_i_ = 220 ± 9.2 nM, 0 nM; Panel B: Δ[Ca^2+^]_i_ = 250 ± 12 nM, 90 ± 8 nM; Panel C: Δ[Ca^2+^]_i_ = 180 ± 10 nM, 165 ± 8 nM; Panel D: Δ[Ca^2+^]_i_ = 200 ± 9.8 nM, 200 ± 12 nM. Panels E and F: HEK.TPα^S145A^ (Panel E) or HEK.TP^S145A,Δ328^ (Panel F) cells were stimulated for 4 min with 1 μM U46619 (Primary stimulation; Panels E and F); thereafter, cells were washed and re-stimulated at 15 min following the primary stimulation with 1 μM U46619 (Secondary stimulation; Panels E and F). Actual mean changes in U46619-induced [Ca^2+^]_i_ mobilizations (nM ± S.E.M.) in response to primary and secondary U46619-stimulations were: Panel E: Δ[Ca^2+^]_i_ = 157 ± 3.8 nM, 37 ± 4.2 nM; Panel F: Δ[Ca^2+^]_i_ = 174 ± 7.7 nM, 170 ± 9.9 nM.

To investigate whether the second messenger-regulated protein kinases (PKs) may play a role in mediating homologous desensitization of TPα, the effects of the PKA and PKC inhibitors H-89 and GF 109203X, respectively, on U46619-induced [Ca^2+^]_i_ mobilization were investigated. Pre-incubation of HEK.TPα or HEK.TP^Δ328^ cells with H-89 had no significant effect on [Ca^2+^]_i_ mobilization in response to their primary or secondary stimulation with U46619 (Fig. 3A and C, respectively). Whilst pre-incubation with GF 109203X had no significant effect on [Ca^2+^]_i_ mobilization by TPα or TP^Δ328^ in response to primary agonist stimulation (Fig. 3B and D, respectively), it partially and significantly impaired desensitization of TPα such that in the presence of GF 109203X, secondary U46619 stimulation of TPα yielded a response of 36% relative to that of its primary response (Compare Fig. 3A and B, primary and secondary stimulations). Consistent with previous reports [50], pre-incubation of HEK.TP^Δ328^ cells with GF 109203X fully impaired agonist-induced desensitization in response to its secondary U46619 stimulation (Compare Fig. 3C versus Fig. 3D, secondary responses).

Hence, it is evident that TP^Δ328^ undergoes partial and transient desensitization that occurs through a GF 109203X-sensitive, PKC mechanism, whilst TPα undergoes almost complete and more sustained desensitization that is also partially sensitive to GF 109203X/PKC inhibition. As TP^Δ328^ is devoid of C-tail residues of both TPα and TPβ, it is evident that its target PKC-sensitive site(s) is located at sites other than within the C-tail domain of TPα or TPβ. In fact through computational analysis of the amino acid sequence of TP^Δ328^ we identified a putative PKC site within the second intracellular loop (IC)_2_ where Ser^145^ represents the predicted phospho-target residue [51]. Moreover, through site-directed mutagenesis of both TP^Δ328^ and TPβ, to generate TP^S145A,Δ328^ and TPβ^S145A^ , respectively, we have previously established that Ser^145^ actually corresponds to the GF 109203X-sensitive, PKC site that accounts for the partial agonist-induced desensitization of both TP^Δ328^ and TPβ [50]. Hence, herein, to investigate if Ser^145^ within the IC_2_ of TPα had any role to play in its homologous desensitization, site-directed mutagenesis was used to generate TPα^S145A^. Primary stimulation of HEK.TPα^S145A^ cells (*K*_d_: 6.1 ± 1.3 nM SQ29,548; *B*_max_: 3.03 ± 0.04 pmol/mg protein; Table 1) with U46619 resulted in efficient [Ca^2+^]_i_ mobilization (Fig. 3E) and IP_3_ generation (data not shown) confirming that the mutation *per se* did not affect the basic ligand binding or signalling properties of TPα^S145A^. However, unlike the wild type TPα (Fig. 3E), pre-stimulation with U46619 did not fully desensitize signalling by TPα^S145A^ such that secondary agonist restimulation yielded a response of 24% relative to the primary response (Fig. 3E). Similar data were obtained when agonist-induced IP_3_ generation was analysed (data not shown). Consistent with previous reports, pre-stimulation of TP^S145A,Δ328^ with U46619 did not desensitize [Ca^2+^]_i_ mobilization in response to its secondary stimulation with U46619 at 15 min following the initial stimulation (Fig. 3F, compare primary and secondary stimulations; *p*= 0.55). Taken together, these data confirm that whilst Ser^145^ within IC_2_ represents the phospho-target residue involved in partial U46619-induced, GF 109203X-sensitive homologous desensitization of TP^Δ328^ [50], similar to that of TPβ, Ser^145^ also plays a minor though significant role in the homologous desensitization of TPα. However, the major determinant(s) of U46619-induced desensitization of TPα signalling appear to be located within its unique C-tail domain and that PKC may also have a partial role in that desensitization.

### 3.2 The role of GRKs in homologous desensitization of TPα

**Fig. 4:**
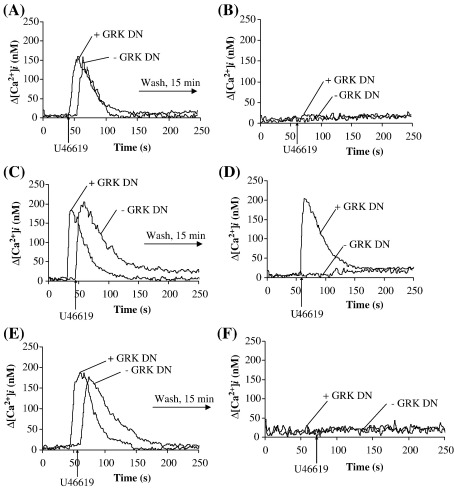
Role of GRK2/3 on U46619-mediated desensitization of signalling by TPα and TPβ. HEK.TPα (Panels A and B), HEK.TPβ (Panels C and D) or HEK.TPα^S239A^ (Panels E and F) cells each transiently co-transfected with either pCMV:Gαq plus pRK5:βARK1^495–689^ (+ GRK^DN^) or, as controls, with pCMV:Gαq plus pRK5 (− GRK^DN^) were stimulated with 1 μM U46619 for 4 min (Panels A, C and E). Thereafter, cells were washed as indicated by the horizontal arrow and re-stimulated at 15 min following the primary stimulation with 1 μM U46619 (Panels B, D and F), where ligands were added at the times indicated by the vertical arrows. Actual mean changes in U46619-induced [Ca^2+^]_i_ mobilization (nM ± S.E.M.) were: Panel A: + GRK^DN^, Δ[Ca^2+^]_i_ = 150 ± 7.5 nM; − GRK^DN^, Δ[Ca^2+^]_i_ = 165 ± 9 nM; Panel B: + GRK^DN^, Δ[Ca^2+^]_i_ = 0 nM; − GRK^DN^, Δ[Ca^2+^]_i_ = 0 nM; Panel C: + GRK^DN^, Δ[Ca^2+^]_i_ = 195 ± 11 nM; − GRK^DN^, Δ[Ca^2+^]_i_ = 180 ± 6.7 nM; Panel D: + GRK^DN^, Δ[Ca^2+^]_i_ = 210 ± 12 nM; − GRK^DN^, Δ[Ca^2+^]_i_ = 0 nM. Panel E: + GRK^DN^, Δ[Ca^2+^]_i_ = 180 ± 9 nM; − GRK^DN^, Δ[Ca^2+^]_i_ = 176 ± 10.3 nM; Panel F: + GRK^DN^, Δ[Ca^2+^]_i_ = 0 nM; − GRK^DN^, Δ[Ca^2+^]_i_ = 0 nM.

In addition to the second messenger-regulated protein kinases, members of the G protein coupled receptor kinase (GRK) family are widely reported to play a central role in homologous desensitization of various GPCRs [36]. Moreover, we have recently established that GRK2/3 plays a critical role in the homologous desensitization of signalling by TPβ [50]. Hence, herein, we investigated the role of the ubiquitously expressed GRK2/3, also referred to as the β adrenergic kinases (β-ARK) 1/2, in the homologous desensitization of signalling by TPα. Initially we examined the effect of over-expressing GRK2/βARK1^495–689^, a dominant-negative form of GRK2/βARK1[39], on U46619-induced desensitization of TPα  comparing it to its effect on TPβ signalling, acting as a reference. Transient over-expression of βARK1^495–689^ had no significant effect on primary U46619-induced [Ca^2+^]_i_ mobilization by TPα or TPβ ^ ^(Fig. 4A and C, respectively). However, while over-expression of βARK1^495–689^ almost fully impaired desensitization of TPβ signalling following its secondary stimulation with U46619 (Compare Fig. 4C versus Fig. 4D, *p* = 0.0001), it had no effect whatsoever on agonist-induced desensitization of signalling by TPα (Compare Fig. 4A versus Fig. 4B). Thus, while GRK2/3 (βARK1/2) play a major role in the homologous desensitization of TPβ they do not appear to participate in desensitization of TPα.

As Ser^239^ has been identified as a critical site in the GRK2/3-mediated homologous desensitization of TPβ [50], a site that is also common to and found within the IC_3_ domain of TPα but is clearly not targeted through GRK2/3 (Fig. 4B), we extended our studies to investigate whether it may have any role to play in homologous desensitization of TPα, such as through phosphorylation by other members of the GRK family or other Ser/Thr kinases. Hence, site directed mutagenesis was used to generate TPα^S239A^, thereby disrupting the phospho-target. Primary stimulation of HEK.TPα^S239A^ cells (*K*_d_: 7.1 ± 3.2 nM SQ29,548; *B*_max_: 4.35 ± 0.04 pmol/mg protein; Table 1) with U46619 resulted in efficient [Ca^2+^]_i_ mobilization (Fig. 4E) and IP_3_ generation (data not shown) confirming that the mutation *per se* did not affect the basic ligand binding or signalling properties of TPα^S239A^. Similar to that of the wild type TPα (Fig. 2A and B), pre-stimulation with U46619 fully desensitized signalling by TPα^S239A^ (Fig. 4E and F). Moreover, over-expression of the dominant negative βARK1^495–689^ had no effect on the primary signalling or on the level of agonist-induced homologous desensitization of TPα^S239A^ (Fig. 4E and F, + GRK DN). These data clearly indicate that while Ser^239^ within IC_3_ is a critical GRK2/3-targeted site in the homologous desensitization of TPβ signalling, that site appears to be redundant in TPα and does not participate in desensitization of signalling of TPα.

### 3.3 Investigation of the role of the unique C-tail domain in homologous desensitization of TPα.

Hence, it is evident that while Ser^145^ within the IC_2_ domain plays a role, at least in part, in the GF 109203X, PKC-dependent homologous desensitization of signalling by TPα, from studies with TP^Δ328^ it appears that the major determinants of TPα desensitization rely on sequences within its unique C-tail domain. Analysis of the C-tail sequence of TPα reveals several Ser/Thr residues that might represent target sites for agonist-induced phosphorylation or desensitization, including Ser^329^, Ser^331^, Thr^337^ and Ser^340^ (Fig. 1). Thus, Ala-scanning and/or deletion mutagenesis was used to disrupt those putative phospho-targets either individually to generate TPα^S329A^, TPα^S331A^, TPα^Δ336^, TPα^T337A^ and TPα^S340A^ or, there after, in various specific combinations as relevant. It is noteworthy that Ser^329^ has previously been identified as a target residue for PKA phosphorylation [33] while Ser^331^ was identified as a target for PKG phosphorylation [35] and Thr^337^ as a target for PKC phosphorylation [32]. Initially, Scatchard analyses confirmed that values obtained for the affinity (*K*_d_) and maximal radioligand binding (*B*_max_) for each of the HEK 293 cell lines stably over-expressing the respective variant receptors compared well to those previously reported for wild type TPα (Table 1) and clearly indicated that the mutations *per se* did not affect their ligand binding properties.

**Fig. 5:**
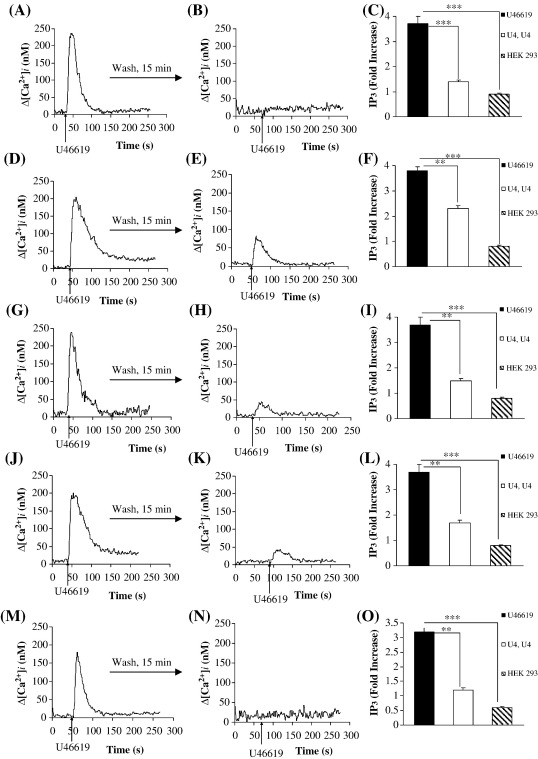
Role of the C-tail domain in agonist-induced desensitization of TPα signalling. Panels A–N: HEK.TPα^S329A^ (Panels A and B), HEK.TPα^S331A^ (Panels D and E), HEK.TPα^Δ336^ (Panels G and H), HEK.TPα^T337A^ (Panels J and K) or HEK.TPα^S340A^ (Panels M and N) cells, each co-transfected with pCMV:Gαq, were stimulated with 1 μM U46619 for 4 min as primary stimulation (Panels A, D, G, J and M). Thereafter, cells were washed to remove the U46619 as indicated by the horizontal arrow and were then re-stimulated 15 min following the primary stimulation with 1 μM U46619 (Panels B, E, H, K and N), where ligands were added at the times indicated by the vertical arrows. Data presented are plotted as changes in intracellular Ca^2+^ mobilization (Δ[Ca^2+^]_i_, nM) as a function of time (second, s). Actual mean changes in U46619-induced [Ca^2+^]_i_ mobilization (nM ± S.E.M.) were as follows: Panel A: Δ[Ca^2+^]_i_ = 228 ± 7.7 nM; Panel B: Δ[Ca^2+^]_i_ = 0 nM; Panel D: Δ[Ca^2+^]_i_ = 207 ± 9.1 nM; Panel E: Δ[Ca^2+^]_i_ = 80 ± 6.8 nM; Panel G: Δ[Ca^2+^]_i_ = 240 ± 14 nM; Panel H: Δ[Ca^2+^]_i_ = 50 ± 3.4 nM, Panel J: Δ[Ca^2+^]_i_ = 200 ± 9.2 nM; Panel K: Δ[Ca^2+^]_i_ ± 49 ± 5 nM, Panel M: Δ[Ca^2+^]_i_ = 180 ± 6.1 nM; Panel N: Δ[Ca^2+^]_i_ ± 0 nM. Alternatively: HEK.TPα^S329A^ (Panel C), HEK.TPα^S331A^ (Panel F), HEK.TPα^Δ336^ (Panel I), HEK.TPα^T337A^ (Panel L), or HEK.TPα^S340A^ (Panel O) cells, co-transfected with pCMV:Gαq, were stimulated with 1 μM U46619 for 2 min (Panels C, F, I, L and O; U46619); cells were then washed and re-stimulated at 60 min following the primary stimulation with 1 μM U46619 for 2 min (Panels C, F, I, L and O; U4, U4). As controls, HEK 293 cells were stimulated for 2 min with 1 μM U46619 (Panels C, F, I, L and O; HEK 293). Levels of IP_3_ produced in ligand-stimulated cells relative to the vehicle (HBS) treated cells (basal IP_3_) were expressed as fold stimulation of basal (fold increase in IP_3_ ± S.E.M.; *n* = 4). The asterisks indicate that the level of U46619-mediated IP_3_ generation was significantly reduced following secondary stimulation compared to that of the primary stimulation, or that the level of U46619-mediated IP_3_ generation was significantly lower in HEK 293 cells than in each of the above cell lines where *, ** and *** indicates *p* < 0.05, *p* < 0.01 and *p* < 0.001, respectively. Basal levels of IP_3_ in the latter cell lines were found to be in the range of 0.27 ± 0.06–0.39 ± 0.09 nmol/mg protein.

Stimulation of HEK.TPα^S329A^ cells yielded efficient [Ca^2+^]_i_ mobilization and IP_3_ generation in response to U46619 (Fig. 5A and C). Moreover, pre-stimulation of HEK.TPα^S329A^ cells with U46619 completely desensitized [Ca^2+^]_i_ mobilization and IP_3_ generation in response to secondary agonist stimulation (Fig. 5A–C). These data concur with previous results using H-89 demonstrating that the PKA inhibitor H-89 had no effect on the homologous desensitization of TPα (Fig. 3A).

Consistent with previous reports [35], agonist stimulation of TPα^S331A^ yielded efficient [Ca^2+^]_i_ mobilization and IP_3_ generation (Fig. 5D and F). Pre-stimulation of HEK.TPα^S331A^ cells with U46619 impaired, but did not fully abolish [Ca^2+^]_i_ mobilization and IP_3_ generation (Fig. 5D–F) in response to secondary stimulation with U46619. Specifically, the level of [Ca^2+^]_i_ mobilization by TPα^S331A^ following secondary stimulation corresponded to 39% of that mobilized following primary U46619 stimulation. These data suggest that Ser^331^ plays a significant role in the homologous desensitization of TPα.

U46619-stimulation of TPα^Δ336^, a truncated variant of TPα eliminating two potential target phosphorylation sites at Thr^337^ and Ser^340^, yielded efficient [Ca^2+^]_i_ mobilization and IP_3_ generation in response to U46619 (Fig. 5G and I, respectively). Pre-stimulation of TPα^Δ336^ with U46619 impaired but did not abolish [Ca^2+^]_i_ mobilization and IP_3_ generation in response to its secondary agonist stimulation (Fig. 5H and I, respectively). Specifically, the level of [Ca^2+^]_i_ mobilization by TPα^Δ336^ in response to secondary U46619 stimulation corresponded to 20% of its response (compare Fig. 5G versus Fig. 5H**,** *p*< 0.0003). Moreover, while both TPα^T337A^ (Fig. 5J and L, respectively) and TPα^S340A^ (Fig. 5M and N, respectively) yielded efficient [Ca^2+^]_i_ mobilization and IP_3_ generation in response to primary stimulation, signalling by TPα^S340A^ was fully desensitized following secondary U46619-stimulation (Fig. 5M–O) while that of TPα^T337A^ was only partially desensitized (Fig. 5J–L). Specifically, the level of [Ca^2+^]_i_ mobilization by TPα^T337A^ in response to secondary stimulation corresponded to 25% of that of its primary response. These data suggest that Thr^337^, a site previously established to act as a PKC site [32], plays a significant role in the homologous desensitization of TPα  and are consistent with findings herein indicating that desensitization of TPα is sensitive to the PKC inhibitor GF 109203X (Fig. 3B).

**Fig. 6:**
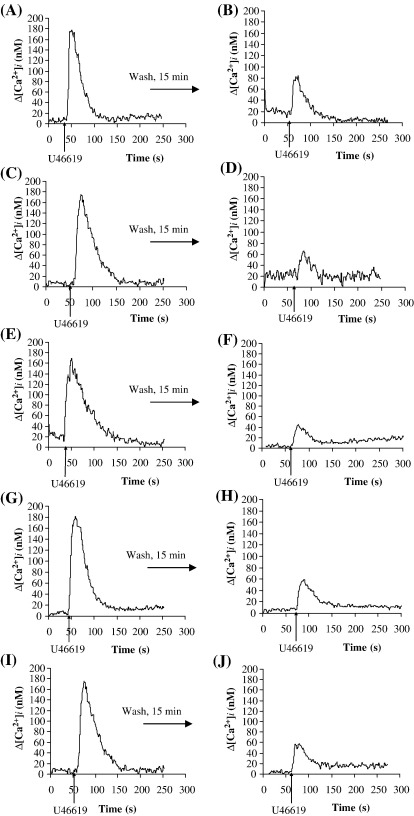
Role of the C-tail domain in agonist-induced desensitization of TPα signalling. Panels A–J: HEK.TPα^S329,331A^ (Panels A and B), HEK.TPα^S331,340A^ (Panels C and D), HEK.TPα^T337,S340A^ (Panels E and F), HEK.TPα^S331,T337A^ (Panels G and H), or HEK.TP^S331,T337,S340A^ (Panels I and J) cells, each co-transfected with pCMV:Gαq, were stimulated with 1 μM U46619 for 4 min as primary stimulation (Panels A, C, E, G and I). Thereafter, cells were washed to remove the U46619 as indicated by the horizontal arrow and were then re-stimulated 15 min following the primary stimulation with 1 μM U46619 (Panels B, D, F, H and J), where ligands were added at the times indicated by the vertical arrows. Data presented are plotted as changes in intracellular Ca^2+^ mobilization (Δ[Ca^2+^]_i_, nM) as a function of time (second, s). Actual mean changes in U46619-induced [Ca^2+^]_i_ mobilization (nM ± S.E.M.) were as follows: Panel A: Δ[Ca^2+^]_i_ = 176 ± 9.4 nM; Panel B: Δ[Ca^2+^]_i_ = 63 ± 4.4 nM; Panel C: Δ[Ca^2+^]_i_ = 160 ± 7.2 nM; Panel D: Δ[Ca^2+^]_i_ = 60 ± 10 nM; Panel E: Δ[Ca^2+^]_i_ = 179 ± 11 nM; Panel F: Δ[Ca^2+^]_i_ = 40 ± 3.4 nM; Panel G: Δ[Ca^2+^]_i_ = 181 ± 6.5 nM; Panel H: Δ[Ca^2+^]_i_ = 60 ± 7.1 nM, Panel I: Δ[Ca^2+^]_i_ = 195 ± 12 nM; Panel J: Δ[Ca^2+^]_i_ = 67 ± 8.2 nM.

Hence, taken collectively, our data generated from the systematic Ala-scanning mutagenesis studies indicate a specific role for Ser^145^ located in IC_2_, Ser^331^ and Thr^337^ located within the C-tail domain in the agonist-induced desensitization of TPα, with no apparent role for Ser^239^ within IC_3_ or Ser^329^ or Ser^340^ in that desensitization. Thereafter, to exclude the possibility that the latter sites might act in concert or in synergy with the former sites in contributing to the desensitization of signalling of TPα, site-directed mutagenesis was used to disrupt those sites in specific combinations (Table 1). Initially we investigated the role of Ser^239^ in combination with Ser^331^ or Thr^337^ to generate TPα^S239,331A^ and TPα^S239,T337A^, respectively. While primary stimulation of TPα^S239,331A^ with U46619 yielded efficient increases in [Ca^2+^]_i_ mobilization and IP_3_ generation (Fig. 6A and data not shown), the level of its agonist-induced desensitization was not significantly different to that of TPα^S331A^ (compare Fig. 6B versus Fig. 5E, respectively). Specifically, the level of [Ca^2+^]_i_ mobilization by TPα^S239,331A^ in response to secondary stimulation corresponded to 36% of the primary U46619 response. Moreover, mutation of Ser^239^ in combination with Thr^337^ to generate TPα^S239,T337A^ did not affect the level of desensitization relative to that of TPα^T337A^ alone (data not shown). These data clearly indicate that mutation of Ser^239^, located within IC_3_, in combination with either Ser^331^ or Thr^337^ does not affect the overall level of TPα desensitization and hence, consistent with our previous data (Fig. 5A–C), we conclude that Ser^239^ does not contribute to TPα desensitization.

Similarly, we also investigated the role of Ser^340^ in combination with Ser^331^ or Thr^337^ to generate TPα^S331,340A^ and TPα^T337,340A^, respectively. While primary stimulation of TPα^S331,340A^ and TPα^T337,340A^ with U46619 yielded efficient increases in [Ca^2+^]_i_ mobilization (Fig. 6C and E, respectively) and IP_3_ generation (data not shown), in each case the level of agonist-induced desensitization was not significantly different to that of their respective single mutants TPα^S331A^ (compare Fig. 6D versus Fig. 5E, respectively) or TPα^T337A^ (compare Fig. 6F versus Fig. 5K, respectively). Collectively these data confirm that mutation of Ser^340^ in combination with either Ser^331^ or Thr^337^ does not affect the overall level of TPα desensitization and hence, consistent with our previous data (Fig. 5M–O), we conclude that Ser^340^ does not contribute to TPα desensitization. Moreover, through a similar series of experiments to generate TPα^S329,331A^ and TPα^S329,T337A^ (data not shown), consistent with our previous data involving TPα^S329^ (Fig. 5A–C) and H-89 (Fig. 3A), we also excluded a role for Ser^329^ in the homologous desensitization of TPα.

Thereafter, we investigated the overall effect of mutating Ser^145^, Ser^331^ and Thr^337^ in combination (Table 1). Stimulation of HEK.TPα^S331,T337A^ cells yielded efficient [Ca^2+^]_i_ mobilization (Fig. 6G) and IP_3_ generation (data not shown) in response to U46619. However, pre-stimulation with agonist only partially desensitized signalling by TPα^S331,T337A^ (Fig. 6G and H). Specifically, the level of [Ca^2+^]_i_ mobilization by TPα^S331,T337A^ in response to secondary stimulation corresponded to 33% of that [Ca^2+^]_i_ mobilized in response to the primary U46619 stimulation. Consistent with these data, the level of agonist-induced desensitization of TPα^S331,T337,S340A^ was some 34% of its primary response (compare Δ[Ca^2+^]_i_ = 195 ± 12 nM for the primary response versus Δ[Ca^2+^]_i_ = 67 ± 8.2 nM for the secondary response; Fig. 6I and J), further confirming a role for Ser^331^ and Thr^337^, but not Ser^340^, in TPα desensitization.

Consistent with the other mutant cell lines, U46619-induced stimulation of HEK.TPα^S145,S331,T337A^ cells, in which Ser^145^ within IC_2_ was also mutated along with Ser^331^ and Thr^337^, yielded efficient [Ca^2+^]_i_ mobilization confirming that the mutation *per se* did not affect its basic signalling. However, pre-stimulation of TPα^S145,331,T337A^ with U46619 only partially desensitized that signalling by such that its level of [Ca^2+^]_i_ mobilization following secondary agonist stimulation corresponded to 55% of its primary response (data not shown). Thus, taken together it appears that TPα is subject to GF 109203X-sensitive desensitization, where Ser^145^ and Thr^337^ [32] within its IC_2_ and C-tail domains, respectively, have been identified as the putative PKC-phospho-targets. In addition, Ser^331^, a site previously identified as a cGMP-dependent PKG site [35] has also been found to be critical for agonist-induced desensitization of TPα.

### 3.4 Agonist-induced phosphorylation of TPα  in whole cells (in vivo)

**Fig. 7:**
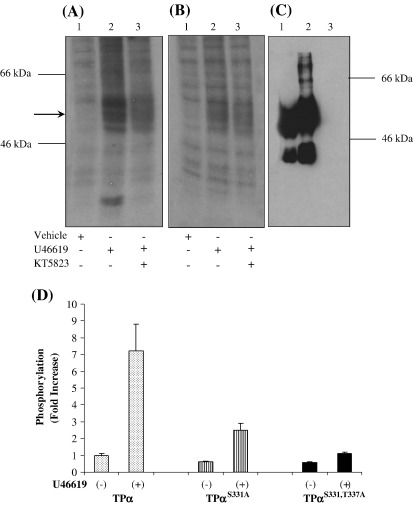
U46619-mediated phosphorylation of TPα. Panels A and B: HEK.TPα (Panel A) and HEK.TPα^S331A^ cells (Panel B), metabolically labelled with [^32^P]orthophosphate, were pre-incubated for 15 min with 50 nM KT 5923 (Panels A and B, lane 3) prior to incubation for 10 min with the vehicle HBS (Panels A and B; lane 1) or 1 μM U46619 (Panels A and B; lanes 2 and 3). Immunoprecipitates were resolved by SDS-PAGE and electroblotted onto PVDF membranes. Blots were subject to PhosphorImage analysis and the intensities of U46619-mediated TPα phosphorylation relative to basal phosphorylation in the presence of HBS were determined and expressed in arbitrary units as follows: TPα: 1 μM U46619, 5-fold increase; 1 μM U46619 plus 50 nM KT 5923, 2-fold; TPα^S331^: 1 μM U46619, 2-fold increase; 1 μM U46619 plus 50 nM KT 5923, 1.5-fold. Panel C: HEK.TPα, HEK.TPα^S331A^ and, as a control, HEK 293 cells (Panel C, lanes 1–3, respectively) were subject to immunoprecipitation with anti-HA antibody 101R and immunoblots screened using the anti-HA 3F10 horseradish peroxidase-conjugated antibody followed by chemiluminescence detection. The positions of the molecular weight markers (kDa) are indicated to the left and right of the panels A and C. The arrow to the left of Panel A indicates the approximate position of the phosphorylated TPα. Data presented are representative of three independent experiments. Panel D: HEK.TPα , HEK.TPα^S331A^ and HEK.TPα^S331,T337A^  cells, metabolically labelled with [^32^P]orthophosphate, were stimulated for 10 min with 1 μM U46619 (+) or with an equivalent volume of the vehicle HBS (−). Thereafter, HA epitope-tagged TPβ receptors were immunoprecipitated as previously described. Blots were subject to PhosphorImage analysis and the intensities of U46619-mediated TPα phosphorylation relative to basal levels in the presence of HBS were determined and expressed in arbitrary units (Phosphorylation; fold increase, *n* = 3).

It has been previously reported that TPα is subject to rapid agonist-induced phosphorylation that is dependent, at least in part, on PKC activation [20,25]. Hence herein, to establish whether TPα undergoes agonist-induced PKG phosphorylation, we investigated the effect of KT 5823 on TPα and TPα^S331A^ phosphorylation in whole cells/*in vivo*. Initially, the specificity of the anti-HA antibodies to immunoprecipitate TPs from the respective cell lines, but not from the parent HEK 293 cells, was confirmed (Fig. 7C). The presence of a discrete band of approximately 39 kDa and a broad diffuse band of 46–60 kDa representing their non-glycosylated and glycosylated forms, respectively, were evident in the TPα and TPα^S331A^ immunoprecipitates (Fig. 7C, lanes 1 and 2, respectively) whilst no immunoreactive bands were evident in immunoprecipitates prepared from non-transfected HEK 293 cells (Fig. 7C, lane 3). Stimulation of HEK.TPα cells  with U46619 led to 5–7-fold increase in the level of TPα phosphorylation relative to vehicle treated cells (Fig. 7A, lanes 1 and 2). Moreover, pre-incubation with KT 5823 reduced the level of agonist-induced TPα phosphorylation by 2.5 fold (Fig. 7A, lane 3). Consistent with this, while stimulation of HEK.TPα^S331A^ cells with U46619 led to 2.5-fold increase in the level of TPα^S331A^ phosphorylation relative to basal levels in vehicle treated cells (Fig. 7B, lanes 1 and 2), agonist-induced phosphorylation of TPα^S331A^ was not significantly affected by preincubation with KT 5823 (Fig. 7B, lanes 1 and 2). Moreover, TPα^S331,T337A^ failed to undergo significant U46619-induced phosphorylation above basal levels (Fig. 7D).

### 3.5 Investigation of the role of Nitric oxide signalling in homologous desensitization of TPα

**Fig. 8:**
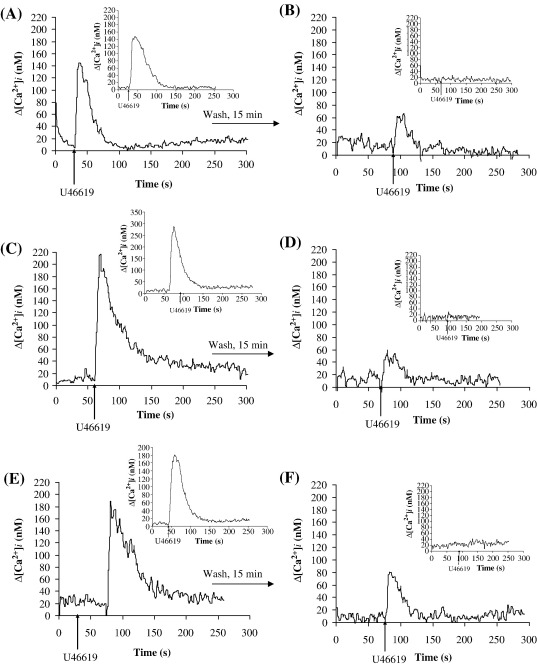
Effect of KT 5823, LY 83583 and l-NAME on U46619-mediated desensitization of TPα signalling. Panels A–F: HEK.TPα (Panels A–F) or, as controls, HEK.TPβ (Insets A–F) cells were pre-incubated for 15 min with either 50 nM KT 5823 (Panels and Insets A and B), 1 μM LY 83583 (Panels and Insets C and D) or 1 μM l-NAME (Panels and Insets E and F) prior to stimulation with 1 μM U46619 (primary stimulation; Panels and Insets A, C and E); cells were then washed to remove the U46619 as indicated by the horizontal arrow and were then re-stimulated at 15 min following the primary stimulation with 1 μM U46619 in the presence of 50 nM KT 5823 (Panels and Insets B), 1 μM LY 83583 (Panels and Insets D) or 1 μM l-NAME (Panels and Insets F). Data presented are representative profiles from at least four independent experiments and are plotted as changes in intracellular Ca^2+^ mobilization (Δ[Ca^2+^]_i_, nM) as a function of time (second, s), where the ligands were added at the times indicated by the arrows. Actual mean changes in U46619-induced [Ca^2+^]_i_ mobilization (nM ± S.E.M.) were: Panel A: Δ[Ca^2+^]_i_ = 160 ± 7.9 nM; Panel B: Δ[Ca^2+^]_i_ = 60 ± 7 nM; Panel C: Δ[Ca^2+^]_i_ = 210 ± 12 nM; Panel D: Δ[Ca^2+^]_i_ = 58 ± 6 nM; Panel E: Δ[Ca^2+^]_i_ = 200 ± 9 nM; Panel F: Δ[Ca^2+^]_i_ = 80 ± 5.9 nM. Inset A: Δ[Ca^2+^]_i_ = 170 ± 25 nM; Inset B: Δ[Ca^2+^]_i_ = 0 nM; Inset C: Δ[Ca^2+^]_i_ = 185 ± 26 nM; Inset D: Δ[Ca^2+^]_i_ = 0 nM; Inset E: Δ[Ca^2+^]_i_ = 220 ± 40 nM; Inset F: Δ[Ca^2+^]_i_ = 0 nM.

To further address whether PKG plays an important role in homologous desensitization of TPα, we examined the effect of various pharmacological inhibitors of the NO/cGMP signalling cascade on U46619-induced desensitization of TPα, and as a control, on TPβ signalling. While pre-incubation of HEK.TPα cells with KT 5823 (50 nM) did not interfere with their ability to mobilize [Ca^2+^]_i_ *per se* (Compare Fig. 2A versus Fig. 8A), it partially inhibited U46619-induced desensitization of TPα signalling in response to secondary stimulation with U46619 (Fig. 8B). Specifically, in the presence of KT 5823, the level of [Ca^2+^]_i_ mobilized by TPα in response to its secondary stimulation with U46619 corresponded to approximately 38% of its primary response (Fig. 8A and B). On the other hand, pre-incubation of HEK.TPβ cells with KT 5823 had no significant effect on U46619-induced [Ca^2+^]_i_ mobilization in response to its primary stimulation and did not impair agonist-induced desensitization of signalling by TPβ (Insets to Fig. 8A and B).

A potent stimulator of PKG is the activation of nitric oxide synthase (NOS) promoting the conversion of l-arginine to nitric oxide. NO, in turn, activates the soluble form of guanylate cyclase (sGC) leading to a rise in intracellular cGMP and activation of PKG [52–55]. Hence, we also investigated the actions of sGC and NOS inhibitors LY 83583 and l-NAME, respectively, on TPα desensitization. While pre-incubation of TPα with LY 83583 did not interfere with its ability to mobilize [Ca^2+^]_i_ *per se* (Compare Fig. 2A versus Fig. 8C), it partially inhibited desensitization of TPα signalling (Fig. 8D). In the presence of LY 83583, the level of [Ca^2+^]_i_ mobilized following secondary stimulation corresponded to approximately 30% of its primary response. LY 83583, on the other hand, had no significant effect on [Ca^2+^]_i_ mobilization by TPβ following its primary or secondary stimulation with U46619 (Insets to Fig. 8C and D). Similarly, the NOS inhibitor l-NAME had no effect on primary U46619-induced signalling by TPα (Compare Fig. 2A versus Fig. 8E) but significantly inhibited desensitization of TPα signalling (Fig. 8E and F). Specifically, in the presence of l-NAME, the level of [Ca^2+^]_i_ mobilized by TPα following secondary U46619 stimulation corresponded to approximately 40% of the primary signal. l-NAME had no significant effect on [Ca^2+^]_i_ mobilization by TPβ following its primary or secondary signalling (Insets to Fig. 8E and F).

**Fig. 9:**
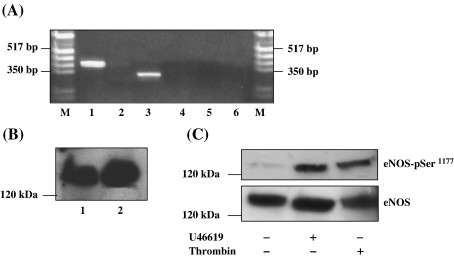
Analysis of nitric oxide synthase (NOS) expression and activation in HEK 293 cells. Panel A: RT-PCR analysis of 1° cDNA from HEK 293 cells as template and primer pairs to selectively amplify neuronal (*n*)NOS (lane 1), endothelial (*e*)NOS (lane 3) or inducible (*i*)NOS (lane 5); negative controls where individual primer pairs were added to the reaction without template cDNA are shown in lanes 2 (*n*NOS), 4 (*e*NOS) and 6 (*i*NOS). Molecular weight markers are shown in lanes M, and the position of the 350- and 517-bp markers are indicated to the left and right of the panel. PCR products corresponding to (*n*)NOS (lane 1; 456 bp) and (*e*)NOS (lane 3; 354 bp) were specifically amplified. Panel B: western blot analysis of eNOS expression. Aliquots (100 μg and 200 μg, respectively) of HEK 293 cell protein was analysed by SDS-PAGE followed by western blot analysis using anti-eNOS antibody. The relative position of the 120-kDA marker is indicated to the left of the panel. Results shown in Panel C: Phosphorylation of eNOS in HEK.TPα cells. HEK.TPα cells were incubated at 37 °C for 5 min with either vehicle, 1 μM U46619 or 10 U/ml Thrombin. Whole cell protein was analysed by SDS-PAGE followed by western blot analysis using anti-phospho eNOS^1177^ and anti-eNOS, as indicated. The relative position of the 120-kDA marker is indicated to the left of the panel. Results shown in Panels A–C are representative of 3 independent experiments.

Thereafter we sought to establish whether any or all of the NOS isoforms are actually expressed in HEK 293 cells. RT-PCR permitted detection of endothelial (*e*)NOS and neuronal (*n*)NOS but not inducible (i)NOS transcripts (Fig. 9A) and western blot analysis also confirmed the abundant endogenous expression of *e*NOS (Fig. 9B) in that cell line. Moreover, consistent with the role of NO/PKG in agonist-induced desensitization of TPα, stimulation of HEK.TPα cells (Fig. 9C) or indeed non-transfected HEK 293 or HEK.TPβ cells (data not shown) with U46619 (1 μM, 5 min) led to the rapid phosphorylation-dependent activation of eNOS, as detected using the anti-phospho eNOS^Ser1177^ antisera (Fig. 9C). In fact the level of eNOS phosphorylation in response to U46619 was comparable, if not greater, than those levels detected in response to stimulation of cells with thrombin (Fig. 9C), an agent that has recently been established to lead to rapid phosphorylation of eNOS at Ser^1177/1179^ leading to its activation [56].

Hence taken collectively, data herein indicate that agonist-induced densensitization of TPα signalling involves two key second messenger regulated systems, one involving PLCβ-regulated PKC activation and phosphorylation of Ser^145^ and Thr^337^ and another involving eNOS phosphorylation and activation leading the NO/cGMP-regulated PKG phosphorylation of Ser^331^. Hence, in addition to the agonist-mediated PKC-feedback mechanism, engagement of the NO-cGMP signalling pathway through U46619-activation of the NOS/sGC/PKG pathway plays a significant role in inducing homologous desensitization of TPα, but not TPβ, in HEK 293 cells.

## 4 Discussion

Agonist-induced homologous desensitization represents a critical regulatory component of the integrated signalling modality of diverse members of the GPCR superfamily and provides a mechanism of terminating, at least in part, the primary signalling response [29,30]. Phosphorylation of the GPCR, typically on Ser/Thr residues within its intracellular loop (IC) or carboxyl-terminal (C) tail domains, initiates such desensitization uncoupling the GPCR from its cognate G-protein(s). The G protein-coupled receptor kinases (GRKs), of which GRK1–7 have been identified, can only phosphorylate the agonist-engaged conformationally active GPCR and hence are typically associated with homologous desensitization [29,30,57]. Recruitment of β-arrestin1/2 adaptor(s) to the GRK-phosphorylated receptor sterically hinders G-protein coupling [30,36,58]. Moreover, depending on the GPCR, β-arrestin recruitment may promote sequestration and internalization of the desensitized receptor into intracellular compartments, such as through clathrin- or caveolin-dependent mechanisms [30,36,58], ultimately leading to either dephosphorylation and recyclization (resensitization), degradation or recruitment of the GPCR into scaffolds/microdomains for participation in other signalling systems [30,36,58–60]. The second messenger-regulated kinases, such as cAMP-dependent protein kinase (PK) A and diacylglycerol-regulated PKC, on the other hand, can phosphorylate GPCRs even in the absence of the specific agonist and, hence, are most frequently associated with heterologous desensitization or cross-talk between different signalling systems, but may also contribute to homologous desensitization in certain cases [29,30].

The platelet-derived prostanoid TXA_2_ regulates a range of physiologic responses mainly within the vasculature and is implicated as a mediator of a host of vascular disorders [3–5,12,13]. TXA_2_ acts as a potent evanescent autocoid with an estimated half-life of 30 s, being rapidly hydrolysed to its inactive metabolite TXB_2_ [1]. However, in such cases as thrombosis, vascular occlusion and hypertension where TXA_2_ is produced in large quantities over prolonged periods [61,62], turning off the TXA_2_ signal may also occur at the level of its receptor TP [29,30,36]. TP also mediates many the actions of the isoprostane 8-*iso*-PGF_2α_ that is generated in abundance in situations of oxidative injury [10,11]. Furthermore, in primates TXA_2_ signals through two TP receptor isoforms, termed TPα and TPβ, adding to the complexity whereby the responses to TXA_2_ and, indeed, 8-*iso*-PGF_2α_ may be regulated in humans. Hence, bearing this in mind, we have recently investigated the mechanisms whereby the cellular responses of TXA_2_ mediated by the TPβ isoform of the human TXA_2_ receptor (TP) are regulated and established that GRK2/3 plays a major role in that homologous desensitization, with an additional minor role for PKC [50]. Specifically, it was found that agonist-induced GRK2/3 phosphorylation of Ser^357^ within the unique C-tail domain of TPβ provides a docking site to recruit and orientate GRK2/3, facilitating its phosphorylation of Ser^239^ within IC_3_, impairing Gq coupling, desensitizing TPβ signalling and promoting its agonist-induced internalization [50]. As TPα and TPβ diverge within their C-tail domains, sequences such as Ser^357^ determined to be critical for desensitization of TPβ are not found in TPα clearly implying that the mechanism of agonist-induced desensitization of the TP isoforms may indeed differ. Moreover, consistent with that hypothesis, TPβ but not TPα undergoes agonist-induced internalization largely through a GRK/β-arrestin-dependent mechanism [37,63]. Hence, the overall aim of the current study was to investigate the molecular mechanism(s) whereby the cellular responses of TXA_2_ mediated by the TPα isoform are specifically regulated thereby aiming to elucidate the mechanism of homologous desensitization of TPα.

It was initially confirmed that, similar to TPβ, TPα was subject to complete agonist-induced homologous desensitization as assessed by measurement of [Ca^2+^]_i_ mobilization and IP_3_ generation in response to the TXA_2_ mimetic U46619. Desensitization of TPα is sustained showing no significant recovery following agonist wash-out and re-stimulation some 60 min following the primary response. On the other hand and consistent with our previous data [50], TP^Δ328^, the truncated derivative devoid of the divergent residues of TPα and TPβ, underwent partial and transient desensitization with its signalling fully recovered to that of its primary response at 60 min. Moreover, desensitization of TP^Δ328^ was blocked by pre-incubation with the PKC inhibitor GF 109203X or by mutation of Ser^145^ within IC_2_ to generate TP^S145A,Δ328^ [50]. While the PKA-inhibitor H-89 had no effect on TPα signalling, GF 109203X quite significantly impaired agonist-induced desensitization such that the magnitude of the secondary U46619-response by TPα was 36% of its primary response in the presence of GF 109203X. Mutation of Ser^145^ within IC_2_ to generate TPα^S145A^, a residue common to both TP isoforms and previously identified as the critical PKC phospho-target of both TP^Δ328^ and TPβ [50], established that the level of desensitization of TPα^S145A^ was partially impaired, signalling to 24% of its primary response following secondary agonist stimulation. Hence, collectively these data suggested that whilst Ser^145^ within IC_2_ represents the PKC phospho-target residue involved in transient and partial desensitization of TP^Δ328^ [50], similar to that of TPβ [50] Ser^145^ also plays a minor though significant role in the homologous desensitization of TPα. However, these data also suggest that the major determinant(s) of desensitization of TPα signalling appear to be located within its unique C-tail domain.

As stated, GRK2/3 plays a major role in both agonist-induced desensitization of signalling and internalization of TPβ where Ser^239^ and Ser^357^ within the IC_3_ and C-tail domains, respectively, were identified as the critical functional targets [50]. Hence, herein, we initially investigated the role of the ubiquitously expressed GRK2/3 in the homologous desensitization of signalling by TPα by examining the effect of over-expressing GRK2/βARK1^495–689^, a dominant negative form that impairs GRK2/3 membrane translocation and activation [39]. While βARK1^495–689^ almost fully impaired desensitization by TPβ, it had no significant effect on primary or secondary signalling by TPα. Moreover, mutation of Ser^239^ within the IC_3_ domain of TPα, a critical GRK2/3-target site within TPβ [50], established that TPα^S239A^ under-went complete agonist-induced desensitization of signalling similar to the wild type TPα. Collectively these data clearly indicate that while Ser^239^ within IC_3_ is a critical GRK2/3-targeted site in TPβ, it appears to be redundant in TPα and does not participate in its homologous desensitization and hence that residue is unlikely to be a target for phosphorylation/desensitization by other GRKs, such as by the ubiquitously expressed GRK5 or GRK6 [64]. It is also noteworthy that the sequences DS^239^E and DS^357^R are identically found in both the human and chimp TPβ sequences but neither phosphotarget sites are found in the single bovine, mouse or rat TP orthologs suggesting that both Ser^239^ and Ser^357^ have co-evolved to confer a common function to the TPβ isoforms in primates and clearly account for the redundant role of Ser^239^, in functional terms, within the TPα primate orthologs. The finding herein that GRK/β-arrestins do not play a significant role in homologous desensitization of signalling by TPα are entirely consistent with reports that TPα does not undergo significant agonist-induced internalization [37].

Hence, while Ser^145^ within the IC_2_ domain plays a role in the GF 109203X-sensitive, PKC-dependent homologous desensitization of signalling by TPα, from studies with TP^Δ328^ it was evident that the major determinants of TPα desensitization rely on sequences within its unique C-tail domain. The C-tail sequence of TPα has several Ser/Thr residues that might represent target sites for agonist-induced phosphorylation or desensitization, including Ser^329^, Ser^331^, Thr^337^ and Ser^340^. Moreover, several of those sites have been identified as targets of heterologous desensitization [32–35]. Hence, Ala-scanning and/or deletion mutagenesis was used to systematically disrupt those putative phospho-targets either individually, to generate TPα^S329A^, TPα^S331A^, TPα^Δ336^, TPα^T337A^ and TPα^S340A^, or in specific combinations as relevant and the functional consequences of those mutations on ligand binding, primary signalling and agonist-induced desensitization were investigated. Through these mutational studies, it was established that neither Ser^329^ nor Ser^340^, mutated either alone or in combination with other putative phospho-target residues, had any role to play in the mediation of homologous desensitization of TPα. It is noteworthy that Ser^329^ of TPα had been previously identified as a target for PKA phosphorylation and heterologous desensitization [33]. Hence, our data herein eliminating a role for Ser^329^ in the homologous desensitization of TPα are in agreement with that study [33] and with the fact that the PKA inhibitor H-89 had no effect on the desensitization of TPα.

On the other hand, mutation of Ser^331^ or Thr^337^ alone or in combination with each other or with Ser^145^ led to partial or near complete impairment of agonist-induced desensitization of TPα. Specifically, the level of [Ca^2+^]_i_ mobilization by TPα^S331A^ and TPα^T337A^ following their secondary stimulation corresponded to 39% and 25% of their primary responses, respectively. Moreover, secondary agonist stimulation of TPα^S331,T337A^ or TPα^S145,S331,T337A^ yielded responses corresponding to 33% and 55% of their primary responses, respectively. The involvement of Thr^337^, a site previously identified as a target for PKC phosphorylation [32], is consistent with a significant role for PKC in the agonist-induced desensitization of TPα and clearly indicates that both Ser^145^ and Thr^337^ within the IC_2_ and C-tail domains are functional PKC targets. In terms of Ser^331^, it was previously established that TPα, but not TPβ, is subject to desensitization in response to the potent vasodilator nitric oxide (NO) that occurs through direct type 1 PKG-mediated phosphorylation where Ser^331^ was identified as the PKG phospho-target [35]. Hence, these studies [35] and data herein suggested a direct role for the NO/PKG-mediated signalling in both the heterologous and homologous desensitization of TPα. Consistent with this, KT 5823, the selective PKG inhibitor, significantly reduced U46619-induced phosphorylation of TPα but had no affect on phosphorylation of TPα^S331A^ or TPα^S331,T337A^. Moreover, pre-incubation with KT 5823 reduced agonist-induced desensitization of signalling by TPα such that in its presence the level of [Ca^2+^]_i_ mobilized following secondary agonist stimulation corresponded to ~ 38% of the primary response. Similarly, the sGC and NOS inhibitors LY 83583 (30%) and l-NAME (40%), respectively, yielded significant impairments in agonist-induced desensitization of TPα. On the other hand, consistent with previous findings that TPβ is not a target for NO-mediated heterologous desensitization or PKG phosphorylation [35], neither KT 5823, LY 83583 nor l-NAME had any measurable effect on TPβ signalling following its primary or secondary agonist stimulation. Phosphorylation of eNOS at Ser^1177 (Human)/1179 (Bovine)^ by phosphatidylinositol 3-kinase (PI3-K)-dependent AKT or, as more recently demonstrated, by Gq/PLCβ/PKCδ pathway, such as in response to vascular endothelial growth factor [65] or thrombin stimulation, respectively [56], plays a critical role in eNOS activation. Herein, RT-PCR and/or western blot analysis confirmed the endogenous expression of both eNOS and nNOS in HEK 293 cells. Moreover, agonist (U46619)-stimulation of HEK.TPα cells, or indeed of non-transfected HEK 293 or HEK.TPβ cells (data not shown), led to the rapid phosphorylation of endogenous eNOS at Ser^1177^ to levels comparable, if not greater, than those in response to thrombin stimulation.

**Fig. 10:**
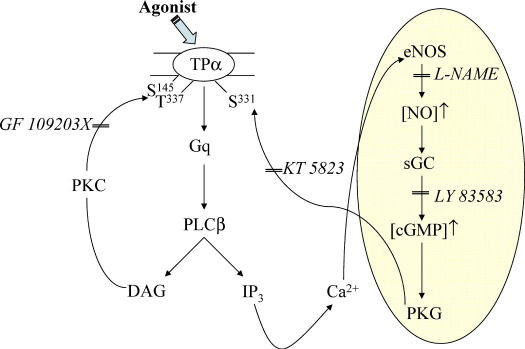
Proposed mechanism of U46619-mediated, homologous desensitization of TPα signalling. Ligand (U46619)-engagement of TPα stimulates Gq-mediated PLCβ activation leading to increases in IP_3_ generation and mobilization of [Ca^2+^]_i_. The latter rise in [Ca^2+^]_i_ triggers activation of endothelial nitric-oxide synthase (eNOS) promoting conversion of l-arginine to nitric oxide (NO). U46619-mediated TPα activation can also lead to increased phosphorylation of eNOS at Ser^1177^, through an as yet unknown mechanism, contributing to eNOS activation (not shown). NO in turn activates the soluble form of guanylyl cyclase (sGC) leading to a rise in intracellular cGMP and, in turn, activation of protein kinase (PK) G. PKG, in turn, phosphorylates and inhibits signalling by TPα where Ser^331^ has been identified as the target residue for PKG phosphorylation. U46619-mediated desensitization of TPα signalling may be inhibited by the NOS inhibitor l-NAME, by the sGC inhibitor LY 83583 or by the PKG inhibitor KT5823. Additionally, TPα/Gαq/PLCβ mediated-increase in DAG leads to activation of PKC; PKC can phosphorylate TPα at Ser^145^ and Thr^337^ yielding partial U46619-mediated desensitization of TPα signalling. Thus, U46619-induced homologous desensitization of TPα is partially mediated through both PKG and PKC-catalysed mechanisms where Ser^331^ and Ser^145^/Thr^337^ have been identified as the respective phospho-targets.

Hence, based on data presented herein, we propose a model for the mechanism of homologous desensitization of TPα signalling, as outlined in Fig. 10. Agonist activation of TPα and TPβ primarily couple to Gq-dependent PLCβ activation leading to increases in IP_3_ generation and mobilization of [Ca^2+^]_i_. This, in turn, triggers the rapid and dynamic activation of Ca^2+^/calmodulin-sensitive NO synthases (NOS), such as through Ser^1177^ phosphorylation and activation of eNOS, promoting the synthesis of NO from l-Arg. NO in turn activates sGC leading to a rise in intracellular cGMP and, in turn, activation of cGMP-dependent PKG, or cGK [54, 55]. Type 1 PKGα/β, in turn, phosphorylates and inhibits signalling by TPα where Ser^331^ represents the phospho-target. As TPβ is not a target of PKG phosphorylation [35], it is not subject to direct desensitization by this mechanism. Additionally, TPα/Gq/PLCβ mediated-activation of PKC can phosphorylate Ser^145^ and Thr^337^ contributing, in part, to the desensitization of TPα signalling. Thus, agonist-induced homologous desensitization of TPα is mediated, at least in part, through both PKG and PKC-catalysed mechanisms where Ser^331^ and Ser^145^/Thr^337^ located within IC_2_ and C-tail domains have been identified as the respective phospho-targets. It is noteworthy that collective mutation of those three sites to generate TPα^S145,S331,T337A^ did not eliminate desensitization of TPα, clearly indicating that other factors may also contribute to agonist-induced desensitization of TPα.

The most striking feature of the proposed mechanism of homologous desensitization of TPα presented herein is that it mainly involves feedback loops whereby the second messenger kinases PKC and PKG activated in response to its primary signalling bring about desensitization through phosphorylation of critical residues within its IC_2_ and C-tail domains. The involvement of PKC in the agonist-induced desensitization of TPα is in keeping with studies by Spurney et al., involving the single murine TP where it was determined that mutation of three putative PKC phospho-Ser residues within its C-tail domain significantly attenuated that desensitization [66]. The endogenous vasodilator NO is critical for the maintenance of normal blood pressure [67–69] and protects the blood vessel wall by inhibiting platelet aggregation, secretion, adhesion and fibrinogen binding to its receptor glycoprotein IIb/IIIa [70]. Many intracellular events involving inositol turnover and Ca^2+^ mobilization, that contributes to vasoconstriction and platelet activation status for example, are regulated by the NO/cGMP signalling cascade, mainly through the direct- or indirect-action of PKG. Amongst the key molecular targets of NO-regulated PKG thus far identified is the IP_3_ receptor [71], thereby inhibiting PLC activation and IP_3_-evoked Ca^2+^ release from intracellular stores [72], IP_3_ receptor-associated cGMP kinase substrate or IRAG [73,74], myosin binding substrate [75] and the vasodilator-stimulated phosphoprotein or VASP [76]. Moreover, in recent studies, it was also established that PKG1α indirectly attenuates signalling by the thrombin/protease-activated receptor-1 (PAR-1) through direct activation of regulator of G-protein signalling-2 (RGS-2), increasing the GTPase activity of Gq [77,78]. Furthermore, as stated, through further recent studies it has been demonstrated that thrombin can directly lead to eNOS phosphorylation at Ser^1177/1179^ in bovine aortic endothelial cells in a mechanism involving Gq/PLCβ/PKCδ phosphorylation and Ca^2+^-dependent activation of eNOS [56]. Whilst our data have indeed confirmed that both thrombin and U46619 can readily lead to eNOS phosphorylation at Ser^1177^ in HEK 293 cells, we have not explored whether that eNOS activation occurs through PKCδ or through the classic PI-3K or other mechanism [56].

Whilst our finding herein that agonist-induced desensitization of TPα entails a closed feedback loop involving eNOS phosphorylation/activation, NO generation and subsequent PKG phosphorylation is a mechanism not typically associated with homologous desensitization of GPCRs in general, it is also in agreement with our previous studies demonstrating that TPα is indeed a direct target of NO/PKG phosphorylation and heterologous desensitization [35]. Moreover, increased NO synthesis and release is known to occur in response to TXA_2_ in other cell/tissue types, such as within the pulmonary microvasculature where the local release of NO acts as a compensatory mechanism in response to the proinflammatory and vasoactive effects of TXA_2_ [79]. NO regulation and synthesis via activation of the Ca^2+^ dependent eNOS or nNOS isozymes can occur in response to a diverse array of agonists that signal through GPCRs, including the widely documented bradykinin and muscarinic acetylcholine receptors signalling pathways, in addition to TP [70,80]. Moreover, direct physical interaction between the bradykinin B2 receptor and eNOS yields a reversible inhibitory complex that is readily dissociated on ligand binding and/or elevations in [Ca^2+^]_i_, thereby providing another counter-regulatory mechanism between GPCR/Ca^2+^- and NOS/cGMP-regulated signalling systems [81]. While we have not been able to find any evidence for a direct interaction between eNOS and TPα, or indeed TPβ, such as through co-immunoprecipitation studies (Reid and Kinsella, unpublished), we cannot rule out the possibility that additional mechanisms other than direct phosphorylation of TPα may contribute to the PKG-, or indeed PKC-, induced desensitization of TPα. For example, it has been demonstrated that PKG1α can be phosphorylated both *in vitro* and *in vivo* by PKC at Thr^58^, generating a partially active kinase that is more sensitive to cGMP levels [82]. Whether TXA_2_-mediated PKC activation contributes to PKG1α activation and TPα phosphorylation/desensitization through the latter mechanism remains to be investigated.

Hence, in summary, it is indeed evident that there are fundamental differences in the mechanisms of homologous desensitization of TPα and TPβ adding further to the increasing lines of evidence that the TP isoforms may indeed have distinct physiologic roles. A critical difference between those two mechanisms is that agonist-mediated desensitization of TPα does not involve its internalization while desensitization of TPβ involves its recruitment to an intracellular compartment(s), making it susceptible to down-regulation or, alternatively, potentially available for participation in other signalling systems [30,36,58–60]. The regulation of TPα signalling provides for a dynamic shut down or desensitization of signalling of the potent autocoid TXA_2_ by the counter-regulatory NO, while TPβ remains unchecked by that mechanism. These findings coupled to the fact that TPα, but not TPβ, is also a direct target of prostacyclin-induced heterologous desensitization [33] provide further evidence for a critical role for TPα in vascular hemostasis whilst the role of TPβ, if any, in that essential physiologic process remains to be defined.

## Figures and Tables

**Table 1:** 

Cell lines	*K*_d_ (nM ± S.E.M.)	*B*_max_ (pmol/mg protein ± S.E.M.)
HEK.TPα^WT^	10.5 ± 1.1	4.52 ± 0.05
HEK.TP^Δ328^	7.2 ± 4.1	4.47 ± 0.04
HEK.TPα^S145A^	6.1 ± 1.3	3.03 ± 0.04
HEK.TP^S145A,Δ328^	6.1 ± 3.7	3.83 ± 0.03
HEK.TPα^S239A^	6.3 ± 2.4	4.35 ± 0.04
HEK.TPα^S329A^	7.1 ± 3.2	4.43 ± 0.04
HEK.TPα^S331A^	6.3 ± 1.2	4.00 ± 0.04
HEK.TPα^Δ336^	7.8 ± 2.1	4.43 ± 0.04
HEK.TPα^T337A^	6.5 ± 1.7	4.23 ± 0.04
HEK.TPα^S340A^	8.1 ± 2.4	5.3 ± 0.04
HEK.TPα^S329,331A^	8.2 ± 1.65	2.9 ± 0.19
HEK.TPα^T337,S340A^	7.5 ± 5	4.9 ± 0.04
HEK.TPα^S331,T337A^	6.9 ± 3.1	3.23 ± 0.03
HEK.TPα^S331,T337,S340A^	6.3 ± 4	4.01 ± 0.05
HEK.TPβ^WT^	9.9 ± 0.5	3.64 ± 0.03

Scatchard analysis of HEK 293 cells stably over expressing HA-epitope tagged forms of TPα or TPβ or their variant receptors were carried out in the presence of the TP antagonist [^3^H]SQ29,548 (50.4 Ci/mmol, 0–40 nM) using 75 μg whole cell protein/assay. Radioligand binding data were analysed with the Graphpad prism V 3.0 computer program (GraphPad Software Inc.) to determine the *K*_d_ and *B*_max_ values. Data are presented as the mean values of four independent experiments ± standard error mean (S.E.M.). HEK 293 control cells expressed 154 ± 4.1 fmol [^3^H]SQ 29,548/mg protein ± S.E.M. (*n* = 3).
